# Effect of acupuncture on tic disorder: a randomized controlled clinical trial based on energy metabolomics and infrared thermography

**DOI:** 10.1186/s12906-024-04534-x

**Published:** 2024-06-20

**Authors:** Yi-ming Fan, Ying-xin Li, Yi Zhang, Dong Chen, Meng-qian Yuan, Yan-cai Li, Elsie Sin May Teo, Ming-hui Xu, Yang-yang Zhou, Pan-feng Yang, Cheng-mei Zhao, Jing-jing Zheng, Jian-bing Li, Chao Bao

**Affiliations:** Jiangsu Province Hospital of Chinese Medicine, Affiliated Hospital of Nanjing University of Chinese Medicine, Nanjing, China

**Keywords:** Acupuncture, Tic disorder, Metabonomics, Infrared thermography, Randomized controlled trial, Study protocol

## Abstract

**Background:**

Acupuncture is a method for treating tic disorder. However, there is a lack of sufficient clinical objective basis in regards of its treatment efficacy. Indeed, there are structural abnormalities present in energy metabolism and infrared thermography in children with tic disorder. Therefore, this study proposes a clinical trial scheme to explore the possible mechanism of acupuncture in treating tic disorder.

**Methods:**

This randomized controlled trial will recruit a total of 90 children, in which they will be divided into non-intervention group and intervention group. The non-intervention group consists of 30 healthy children while the intervention group consists of 60 children with tic disorder. The intervention group will be randomly allocated into either the treatment group or the control group, with 30 children randomly assigned in each group. Children either received acupuncture treatment and behavioral therapy (treatment group) or sham acupuncture treatment and behavioral therapy (control group), 3 treatment sessions per week for a period of 12 weeks, with a total of 36 treatment sessions. Outcome measures include YGTSS, urinary and fecal metabolomics, infrared thermography of body surface including governor vessel. For the intervention group, these outcome measures will be collected at the baseline and 90th day prior to intervention. Whereas for the non-intervention group, outcome measures (excluding YGTSS) will be collected at the baseline.

**Discussion:**

The main outcome will be to observe the changes of the severity of tic condition, the secondary outcome will be to observe the changes of structural characteristic of infrared thermography of body surface/acupoints along the governor vessel and to evaluate the changes of urinary and fecal metabolomics at the end of the treatment, so as to analyze the relationship between them and to provide further knowledge in understanding the possible mechanism of acupuncture in improving the clinical symptoms via regulating and restoring the body metabolomics network, which in future it can develop as a set of clinical guideline (diagnosis, treatment, assessment, prognosis) in treating tic disorder. ChiCTR2300075188(Chinese Clinical Trial Registry, http://www.chictr.org.cn, registered on 29 August 2023).

**Supplementary Information:**

The online version contains supplementary material available at 10.1186/s12906-024-04534-x.

## Introduction

Tic disorder (TD) refers to a kind of neuropsychiatric disorder that usually originated in children or young adults. The main clinical features include involuntary, repetitive, sudden, rapid and non-rhythmic movement of one or more parts of the body and may accompanied with vocal twitch [1]. According to foreign research statistics, the prevalence rate of TD in minors aged 5–18 is 0.4–3.8% [2], and the overall prevalence rate is 1%. It has been reported that about 3% of school-age children have history of transient TD, and 80–90% of TD patients have at least one mental disorder [3]. Furthermore, the symptoms of TD can progress into adulthood, which may lead to employment issues or partner/marriage conflict, which seriously affect one’s quality of life. The neuropathological mechanism of TD is still unclear [4]. At present, it is thought that its occurrence may be related to the content of dopamine in substantia nigra, neuroinflammatory reaction and immune reaction etc [5]. Currently, the clinical diagnosis of TD is mainly based on the symptoms itself, and due to lack of specific biomarker, TD is easy to be misdiagnosed, causing children to lose the opportunity to receive treatment in the golden period. In modern western medicine, a combination of behavioral therapy and drug therapy is usually applied to treat patients with TD. The existing first-line drugs in clinical practice normally require patients to consume it for a long period of time, and the long-term efficacy of the drugs is uncertain, moreover there are various side effects present while consuming it such as nausea, vomiting, headache, insomnia, anxiety and irritability etc [2]. .Acupuncture has been proved to be effective in treating TD, however, the pre-clinical diagnosis is mainly based on scale evaluation [6], and there is a lack of objective evaluation indicators and detection methods.

Energy metabolism may play an important breakthrough in patients with TD. Studies have found that there are differences in neurotransmitters in urine between children with TD and healthy children. Animal models of TD were presented with neurotransmitter abnormalities in cerebrospinal fluid (CSF), detection of neurotransmitters in urine, response to drug therapy that blocks or stimulates neurotransmitters, autopsy findings and neuroimaging studies, which mainly involving dopaminergic, glutamatergic and 5- hydroxytryptamine neurotransmitters [7, 8]. The brain-gut axis is closely related to the occurrence of neuropsychiatric diseases [9]. TD is a chronic neurological disorder. After various clinical observation and animal experiments, the changes of intestinal microflora in patients with TD seem to be specific [10]. The methods of utilizing probiotics and fecal microbiota transplantation in the treatment of TD have shown promising outcomes [11]. The relationship between intestinal microflora and TD as well as the improvement of TD by improving intestinal microflora is rather a new topic, and larger-scale clinical trials are needed to further confirm the influence of intestinal microflora in TD. Therefore, metabonomics detection of urine and feces is beneficial to further study the pathogenesis of TD.

Metabonomics can detect and analyze patients’ urine, feces and other samples through liquid chromatography-mass spectrometry (LC-MS), and can screen hundreds of metabolites on a large scale [12].Modern scholars have found more than 50 kinds of related biomarkers through metabonomics detection of urine in children with TD [13], including p-hydroxyphenylacetic acid, 1- methylhistidine, 4- pyridoxylic acid, etc., The differential markers mainly involve amino acid metabolism, biosynthesis of unsaturated fatty acid, steroid hormone metabolism and other metabolic pathways.Through the screening of fecal metabonomics [14], some scholars have also proved that Tuina combined with acupuncture stimulation of relevant acupoints can improve the relative abundance of Firmicutes, reduce the relative abundance of Proteobacteria at Phylum level, optimize the structure of intestinal microflora and alleviate or treat symptoms of TD. Our research group conducted preliminary clinical studies and found that there are differences in serum amino acid metabolism between children with brain disorders and healthy children [15]. Therefore, this topic intends to conduct a broad-spectrum screening of urine and feces samples of children with TD by using metabonomics technology to determine their differential metabolites, and then refer to KEGG pathway to explore their possible pathogenesis and provide them with more objective biological indicators.

**Fig. 1:**
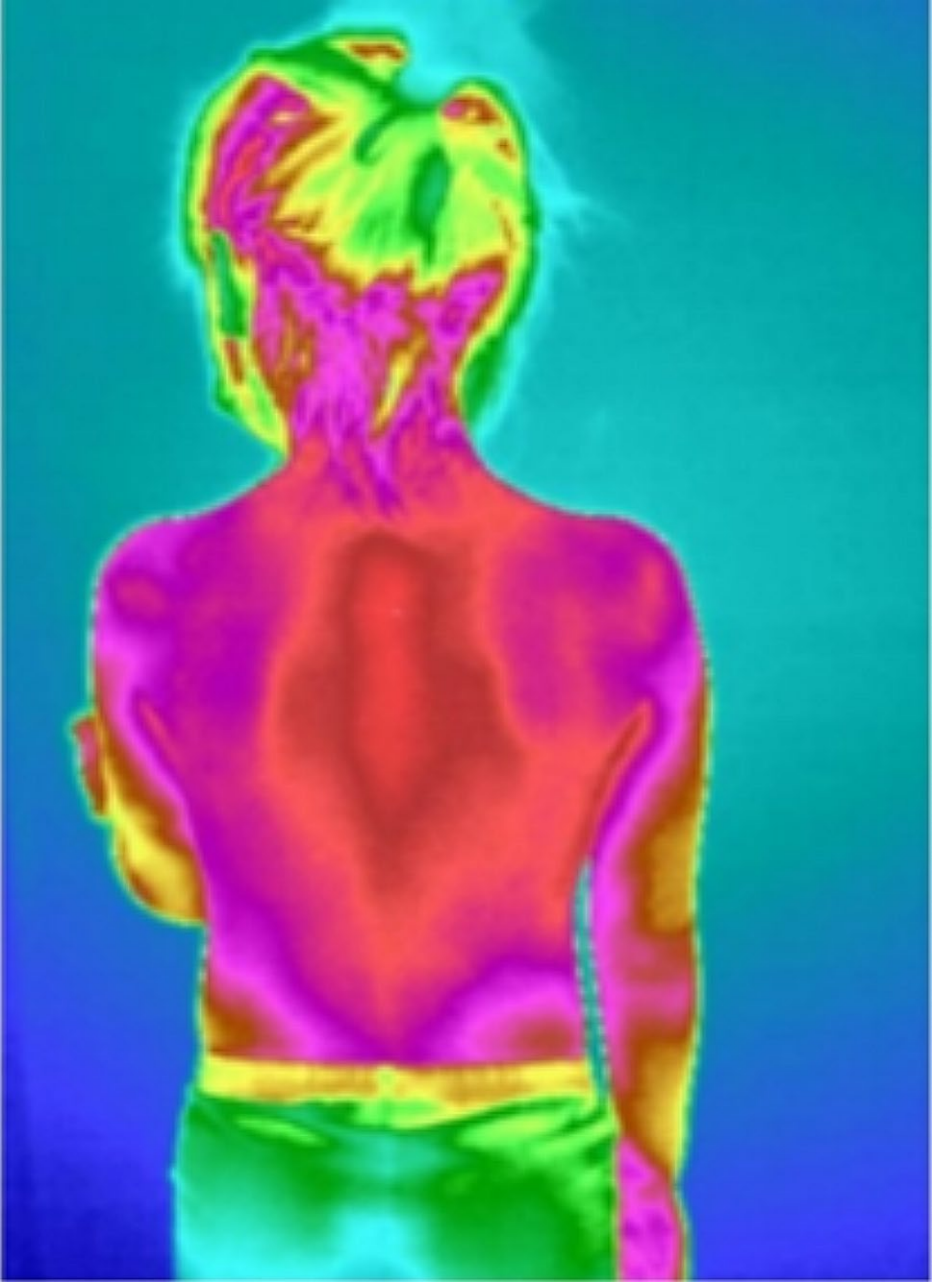
Temperature distribution in the back region of healthy children. In healthy children, a uniform distribution of heat can be observed in the back region (Fig. 1)

**Fig. 2:**
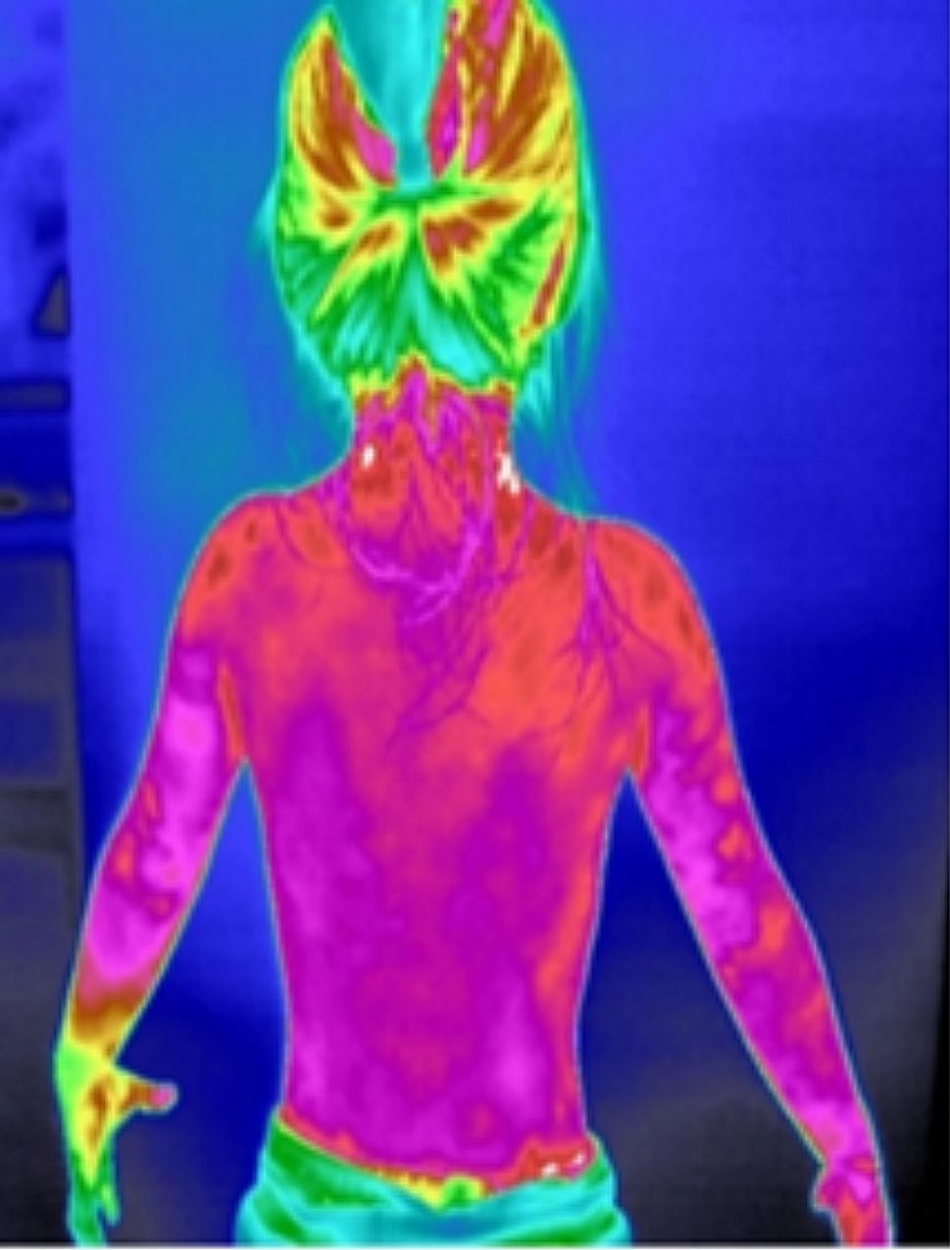
Temperature distribution in the back region of TD children. In the back region of TD patients, there is a tendency for heat to rise (Fig. 2)

The medical infrared imaging system (TTM) can detect the specific band of infrared signals of thermal radiation on the body surface, and convert the signal into images that can be distinguished by human vision, reflecting the thermal structure characteristics of the human body [16]. When a certain part/area of the body happened to be experience pathological changes, the part/area around it will first have changes in tissue metabolism that corresponding to certain pathological manifestation which reflected as abnormal temperature of the skin [17].The mediators involved in regulation of body temperature mainly include PGE2, Na+/Ca2+, corticotropin-releasing hormone (CRH), cyclic adenosine monophosphate (cAMP), NO, etc [18]. When there is a change of endogenous substances in the body, these mediators will be affected, reflected as a change in body temperature. Therefore, we hypothesize that children with TD with abnormal metabolites will also experiencing changes in body temperature. In the early stage of our research, we found that indeed there are differences in the structure of the infrared thermography along the governor vessel between healthy children and children with TD. The heat distribution along the governor vessel in healthy children appeared to be in a uniform state (Fig. 1), whereas in children with TD, the heat distribution along the governor vessel shows a tendency of rising/floating (Fig. 2).

Combined with the abnormality of metabolic substances in children with TD, we speculate that this metabolic abnormality may be related to the changes of structure of infrared thermography of specific areas along the governor vessel and acupoints around it. Therefore, we aim to investigate the mechanism of acupuncture in regulating the energy metabolism in children with TD, and to further explore the underlying molecular mechanism by which changes of body temperature occur, and to assist in screening out microscopic biomarkers of energy groups related to treatment efficacy, as well as to provide theoretical and experimental basis of infrared thermography technology in diagnosing TD.

**Fig. 3:**
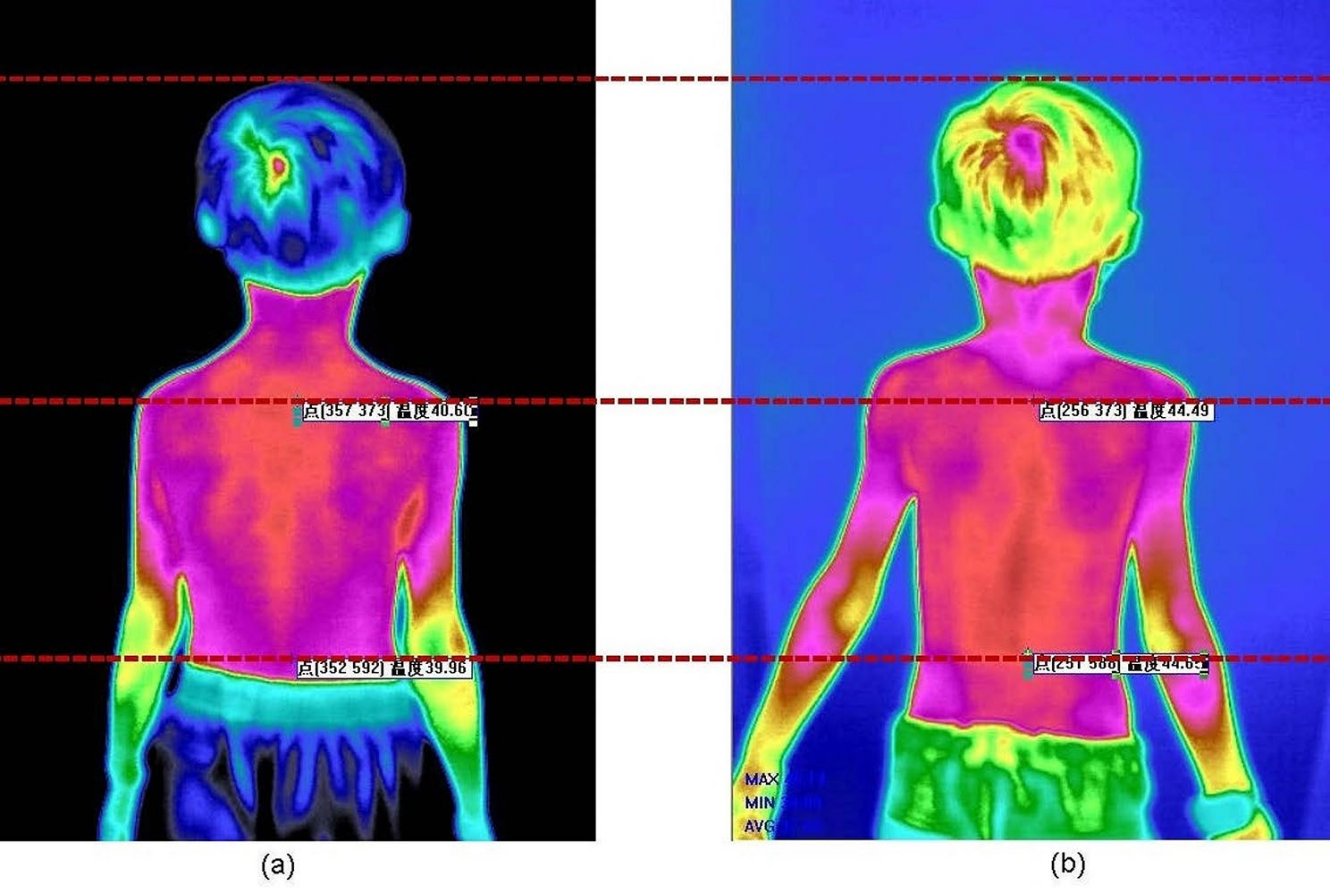
Infrared thermographic changes in the back of TD patients. The thermal distribution on the back before and after acupuncture treatment in individuals with TD is presented from left to right

Our team’s previous research has confirmed the significant therapeutic effects of acupuncture in relieving symptoms associated with tic disorders in children [19]. Preliminary findings of our studies show that the distribution of infrared thermography along the governor vessel in children with TD have significant changes (Fig. 3). At the same time, we also prove that acupuncture can change the types and contents of serum amino acids in children with encephalopathy [15], and most of these amino acids are involved in energy metabolism [20].

Therefore, we designed this randomized controlled trial to compare the changes of the severity of tic condition before and after the intervention, and to observe the changes of structural characteristic of infrared thermography of body surface/acupoints along the governor vessel and to evaluate the changes of urinary and fecal metabolomics at the end of the treatment. By comparing both the non-intervention group and intervention group, we hope to analyze the relationship between them and to provide further knowledge in understanding the possible mechanism of acupuncture in improving the clinical symptoms of TD via regulating and restoring the body metabolomics network.

## Materials and methods

### Study design and participants

**Fig. 4:**
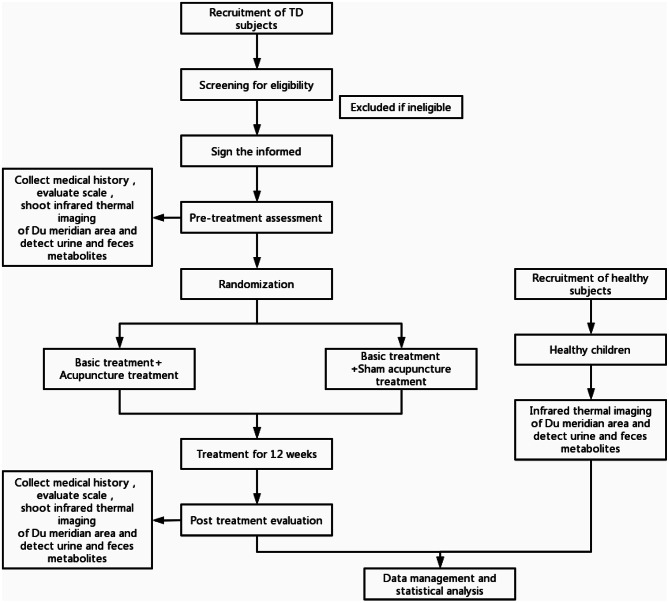
Flow chart. Flow chart of the trial

This is a randomized controlled trial, recruiting a total of 90 children (30 healthy children and 60 children with TD) in the outpatient department of acupuncture and rehabilitation department of Affiliated Hospital of Nanjing University of Traditional Chinese Medicine, in which they will be divided into non-intervention group and intervention group. The non-intervention group consists of 30 healthy children while the intervention group consists of 60 children with TD. The intervention group will be randomly allocated into either the treatment group or the control group, with 30 children randomly assigned in each group. Participants with tic disorder will also be recruited through posters and official WeChat accounts. The researchers will use the American Psychiatric Association DSM-5 criteria for tic disorder to assign a diagnosis. Individuals who meet the inclusion criteria will receive free complimentary clinical testing and some treatment subsidies to encourage enrollment. The details of the clinical trial and the collection of samples will be explained to the parents of the patients before obtaining their written consent. This research was approved by the Ethics Committee of the Affiliated Hospital of Nanjing University of Traditional Chinese Medicine (Jiangsu Provincial Hospital of Traditional Chinese Medicine) (2023NL-095-02), and the trial has been registered in China Clinical Trial Registration Center(ChiCTR2300075188).The flow of the trial is presented in Fig. 4. The schedule of the trial is shown in Table 1.

**Table 1:** The schedule of the trial

Time point	Study period			
	Baseline		Treatment phase	
	Week − 1	Week 0	Week 1	Week 12
Eligibility Screen	×			
Informed consent	×			
Medical history		×		
Random allocation		×		
Intervention				
Acupuncture group (*n* = 30)			×	×
Sham acupuncture group (*n* = 30)			×	×
Primary outcome	YGTSS	×		×
Secondary outcomes	Urine and feces metabonomics	×		×
	Infrared thermography of du meridian area	×		×
Assessments			×	×
Adverse events			×	×
Comprehensive curative effect evaluation			×	×
Safety evaluation			×	×

### Case criteria

This study commenced in September 2023 and is anticipated to be completed by December 2025. Sixty children diagnosed with tic disorder from the Affiliated Hospital of Nanjing University of Traditional Chinese Medicine will be randomly assigned to either the treatment group or the control group, with 30 children in each group. Additionally, 30 healthy children will be recruited for comparison purposes.

#### Inclusion criteria

(1) Participants must meet the diagnostic criteria for TD as according to the American Diagnostic and Statistical Manual of Mental Disorders, 5th edition (DSM-5); (2) Age between 3 and 14 years old; (3) Have stopped using other drugs for mental illness for more than 3 months before starting the treatment; (4) Self-volunteer as the subject, signed by the child’s guardian, and children aged 8 and above get their own informed consent.

#### Exclusion criteria

(1) TD caused by chorea, hand and foot thrombosis, hepatolenticular degeneration, encephalitis and drug-induced; (2) Patients with mental illness; (3) People with mental retardation; (4) Patients with cardiovascular, liver, kidney and hematopoietic diseases.

#### Drop-out criteria

(1) Those who experience severe adverse reactions after acupuncture. (2) Co-authors who drop out mid-way. (3) Previously received a sufficient course of acupuncture technique rehabilitation intervention.

### Sample size

Currently, the overall incidence of Tic Disorder (TD) is approximately 1%. Based on preliminary test results from our research group [21], the average effectiveness rate of the acupuncture intervention group is 95%, while that of the control group is 55%. Accounting for a dropout rate of 20%, a total of 60 cases, with 30 cases in each group, were determined using the sample size calculation formula.

### Randomization

The subjects will be randomly assigned to two groups: the experimental group and the control group. There will be 30 cases in each group. Randomization was performed using the R software, generating a random number table with a block length of 4 and a distribution ratio of 1:1. Upon enrollment of eligible subjects, the randomizer allocated them to groups based on the order of their entry into the trial, labeling “1” as the experimental group and “2” as the control group. Throughout the trial, the acupuncturist will be aware of the patient’s group allocation. The subjects, evaluators of therapeutic effects, and individuals conducting the statistical analysts will be blinded to group allocation.

## Intervention

To ensure optimal therapeutic outcomes, all acupuncture treatments are conducted by a qualified acupuncturist with over 3 years of clinical experiences. Prior to the trial, the acupuncturist undergoes specialized training on the treatment process and adheres to standardized acupuncture protocols.

### Behavior therapy

Psychological counseling is provided to children and their families, offering information about the disease and assisting in developing a proper understanding of Tic Disorder (TD). The importance of a nurturing living environment and harmonious interpersonal relationships for the child’s recovery is emphasized. Encouraging children to engage in communication with their peers helps alleviate any inferiority complexes that they may have, while guiding them to actively participate in creative activities which aims to divert their attention and reduce the occurrence of TD symptoms, thus facilitating their rehabilitation. Additionally, families are advised to plan a healthy balanced diet and creating a well-structured routine to ensure consistency and alleviate academic pressures on children [22].

### Acupuncture needles

Acupuncture needle: The disposable stainless steel acupuncture needle used in this study is the “Huatuo” brand, with dimensions of 0.30 mm in diameter and 25 mm in length, manufactured by Suzhou Medical Supplies Factory.

**Fig. 5:**
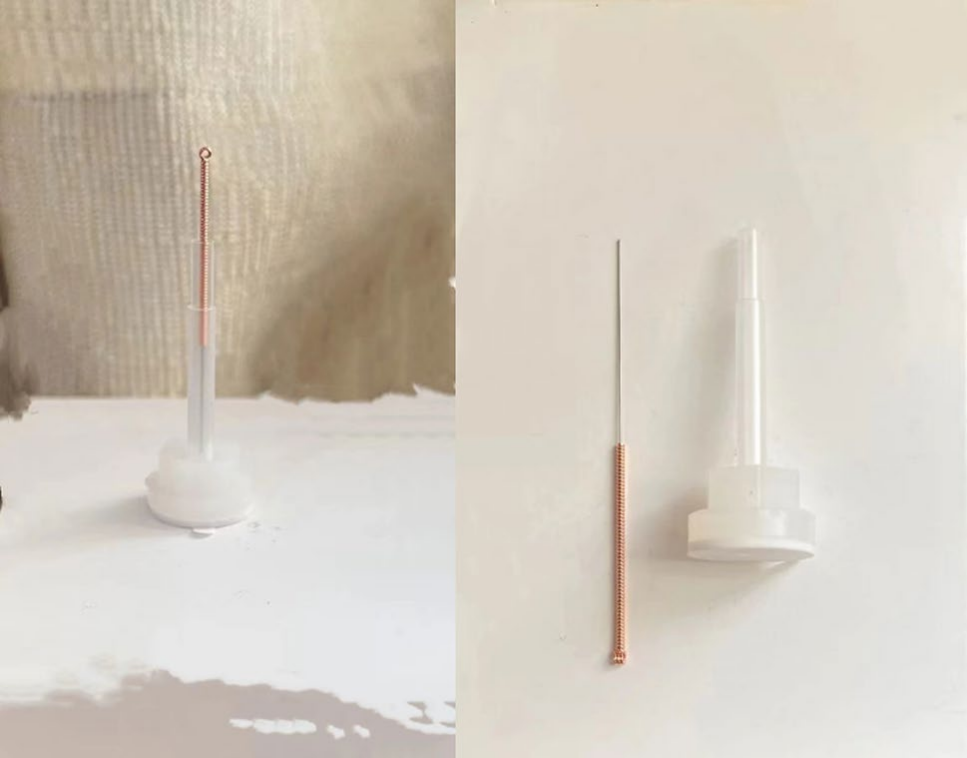
Sham acupuncture needles

Sham acupuncture device: The PSD + retractable blunt needle, a disposable stainless steel sterile blunt needle imported from Britain and produced by Acu Prime brand, was employed in this study. The selected specifications for this needle are 0.30 mm in diameter and 25 mm in length. It is authorized by Dong Bang Acupuncture Company, with approval number 9,018,390,000. (Fig. 5)

### The experimental group

**Fig. 6:**
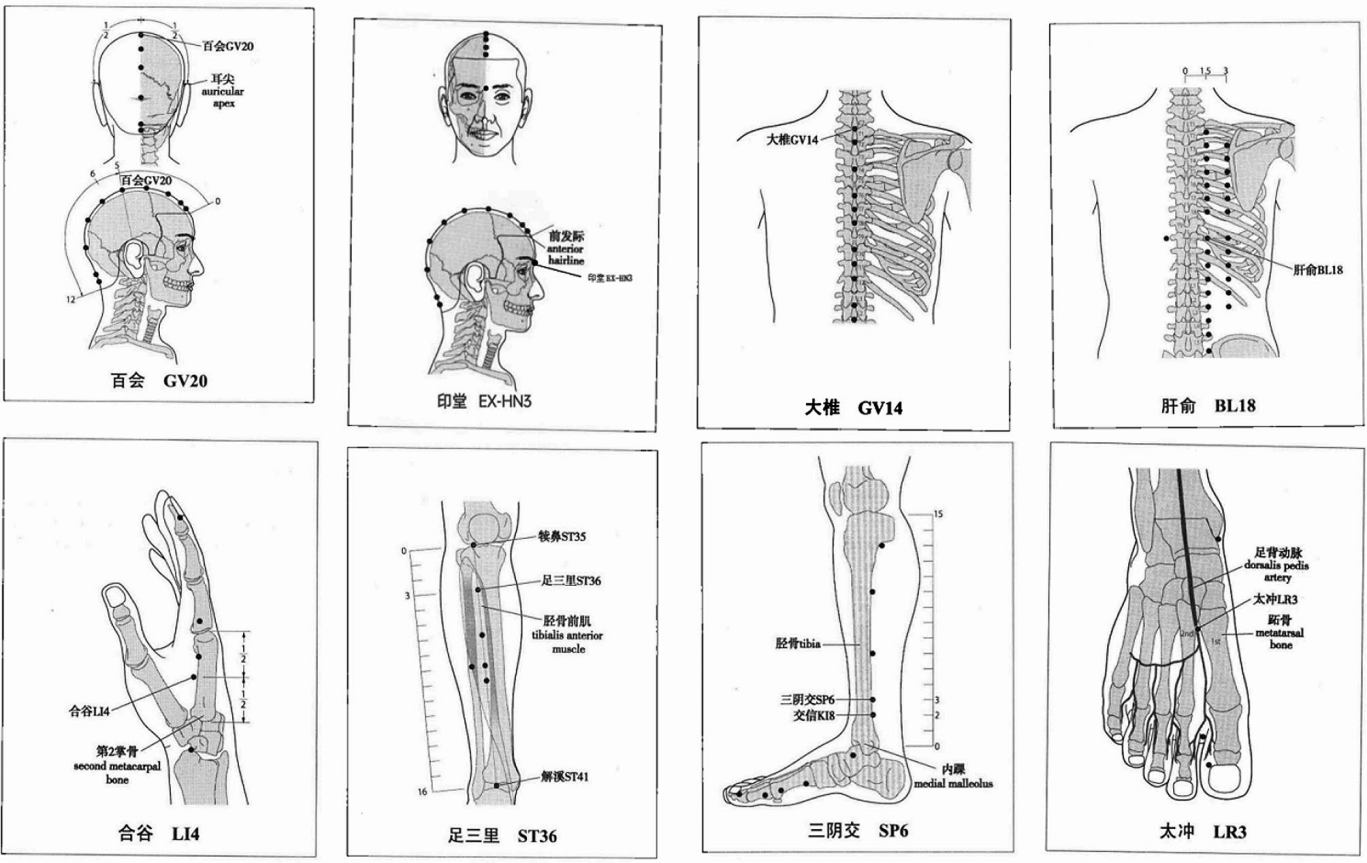
Acupuncture point. The accurate positioning of acupuncture points

The experimental group received a combination of acupuncture therapy and behavioral therapy. The acupuncture protocol consisted of targeting specific acupoints including Baihui, Yintang, Dazhui, Ganshu, Hegu, Zusanli, Sanyinjiao, and Taichong. For the precise location of these acupoints, please refer to Table 2; Fig. 6.

**Table 2:** The precise location of the acupoints

Acupuncture points	Description
GV20: Baihui	On the head, 5 B-cun superior to the anterior hairline, on the anterior median line.
EX-HN3: Yintang	On the forehead, at the midpoint between bilateral eyebrows.
GV14: Dazhui	In the posterior region of the neck, in the depression inferior to the spinous process of the seventh cervical vertebra (C7), on the posterior median line.
BL18: Ganshu	In the upper back region, at the same level as the inferior border of the spinous process of the ninth thoracic vertebra (T9), 1.5 B-cun lateral to the posterior median line.
LI4: Hegu	On the dorsum of the hand, radial to the midpoint of the second metacarpal bone.
LR3: Taichong	On the dorsum of the foot, between the first and second metatarsal bones, in the depression distal to the junction of the bases of the two bones, over the dorsalis pedis artery.
ST36: Zusanli	On the anterior aspect of the leg, on the line connecting ST35 with ST41, 3 B-cun inferior to ST35.
SP6: Sanyinjiao	On the tibial aspect of the leg, posteriorto the medial border of the tibia, 3 B-cun superior to the prominence of the medial malleolus.

The therapeutic needle selected for this study is the disposable stainless-steel needle (0.30 mm in diameter and 25 mm in length), produced by the “Huatuo” brand of Suzhou Medical Supplies Factory. Following routine skin disinfection, the acupuncture angle and depth will be adjusted based on the specific requirements of each acupoint. Subsequently, the electroacupuncture device is connected to both sides of Hegu and Taichong; as on head Baihui and Yintang is connected as a pair, by utilizing a continuous wave frequency at a voltage in which the child can tolerate. Participants will receive three 30-minutes acupuncture sessions per week for a total of 12 weeks.

### The control group

This group received a combination of sham acupuncture and behavioral therapy, and the acupuncture points selected are the same as used in the experimental group. Remove the apron on the skin surface of a PSD base, introduce a disposable blunt needle, expose the needle tip, stick it on the acupoint. Since the blunt needle will retract back into the hollow handle of the needle when it touches the skin, it will not penetrate through the skin and so the needles will just appear to be floating on the skin surface. Acupuncture manipulation, electroacupuncture treatment, treatment duration and period are the same as those in the experimental group.

All acupuncture treatments conform to the Report Standard of Intervention Measures in Acupuncture Clinical Trials [23], refer to supplement 2.

### The healthy group

The healthy group will not take any treatment.

## Outcome evaluations

### Primary outcomes

Yale Comprehensive Tic Severity Scale (YGTSS) is the most commonly used scale to evaluate the severity of tic condition in clinic. The scale scores motor tic and vocal tic respectively, and clinicians score them by asking questions from the guardians and observing their performance in clinic. The total score of YGTSS in patients with TD, if it is less than a score of 25, it is considered as mild, a score of 25 to 50 is considered as moderate, and a score of 50 above is considered as severe.

### Secondary outcomes

#### Infrared image acquisition at the back along the governor vessel

Infrared images will be captured along the governor vessel of both groups of children: Healthy children and children with TD, aiming to identify any dissimilarities between these two groups. Additionally, infrared images will be captured in children with TD before and after the intervention, as for a comparative analysis.

#### Metabolomics

Fecal and urine samples will be collected from both groups of children: healthy children and children with TD. Through analysis and detection, biomarkers associated with acupuncture treatment in children with TD can be discovered. Additionally, the KEGG database will be utilized to analyze the metabolic pathways linked to these biomarkers in the acupuncture treatment of children with TD.

## Infrared thermography system capture

### Research instrument

The BK-MT02A digital medical infrared thermal imager, manufactured by Beijing Zhongrui Bokang Medical Equipment Co., Ltd., will be utilized to capture images of the spinal region.

### Testing requirements

The environment of the infrared thermography examination room is relatively stable, the temperature is maintained at 24 degrees Celsius, the relative humidity is less than 70%, the indoor air flows naturally, there is no wind and dust, and there is no direct heat source. Subjects should avoid drinking alcohol, coffee and other irritating foods 24 h before shooting, prohibit from eating too cold or overheated food 1 h before the shooting, avoid strong cold or overheated environment (such as direct blowing under air conditioning), and stop physical therapy and taking drugs 10 min before shooting and avoid leaning on object.

After entering the examination room, the subject should take off his/her shirt and expose the bare shooting area, remove necklaces and other accessories, and hair should not cover the forehead and neck, the subject is required to sit quietly and rest for 15 min to adapt to the ambient temperature, and avoid pressing or scratching the examination area during the rest period.

### Acupoint examination

The anatomical coordinates of the Dazhui, Zhiyang, Mingmen, Yaoyangguan, and Jinsuo points on the body surface were determined. To facilitate the localization of these acupoints during image acquisition without altering their local body surface temperature, a custom-made hollow foam adhesive cube, measuring 14 mm in inner diameter and 3 mm in height, is applied as an insulating marker on each acupoint.

### Testing process

During the shooting process, the subject keeps the body upright, facing the camera with both upper limbs hanging down on both sides of the body. The infrared camera lens is 1 m to 2 m away from the subject, and the professional technician will take the picture, collect the infrared thermogram of the spine area and save the infrared image. Within the Du meridian area, specific meridian points such as Dazhui, Zhiyang, Mingmen, Yaoyangguan, and Jingsuo, will be manually traced. Afterwards, the computer automatically measure the average temperature of this area, record as t, and document the inspection results.

## Metabolomics Research

### Establishment of TD-related Metabonomics Database

We conducted a comprehensive search across several databases and documents, including HMDB, Medline, NCBI, Web of Science, Wanfang Database, China Journal Network, and etc., to investigate the metabolic process and potential metabolites associated with this disease. This findings serve as a valuable reference for sample processing, detection, and analysis.

### Sample collection and processing

#### Sample collection

##### Urine sample

Prior to sample collection, it is essential to wash your hands thoroughly. Collect a urine sample larger than 1 mL and store it at a temperature of 4 °C. Allow the sample to stand for a period of time. Following this, centrifuge 1 mL of the urine at a temperature of 4 °C, with the settings at 10,000 rpm for 5 min. Absorb 100 µL of the supernatant and transfer it into appropriately numbered EP tubes. Repeat this process, subpackaging the sample into multiple tubes to prevent repeated freezing and thawing. Finally, rapidly freeze the tubes in liquid nitrogen and store them in a refrigerator at -80 °C.

##### Fecal sample

To begin, prepare the feces container, wash your hands thoroughly, and wear gloves. Use sterile spoons, provided with the sampling tube cover, to collect 2–3 spoons of fecal samples for each tube. Once collected, immediately place the samples in low-temperature conditions such as liquid nitrogen, dry ice, ice bags, or a refrigerator set at 4 degrees Celsius. Subsequently, promptly transfer the samples to a -80-degree refrigerator for long-term storage.

#### Sample pretreatment

Urine: Thaw the stored urine at 4℃, shake and mix well, take 500µL and transfer it to a 1.5 mL EP tube, centrifuge at 12,000 rpm for 10 min at 4℃ to remove sediment, take 200µL of the supernatant and transfer it to another 1.5 mL EP tube, add 200µL of methanol, vortex for 3 min, centrifuge at 12,000 rpm for 10 min at 4℃, collect the supernatant for analysis.

Fecal: Add the sample and n-hexanoic acid to sterile distilled water, mix well, then add 0.4 ml of sulfuric acid (approximately 50% mass fraction) and 2 ml of anhydrous ether. Place the mixture on a shaker and mix for 30 min, followed by centrifugation at 3000r/min. Add anhydrous CaCl2 to remove residual moisture, and transfer the supernatant for GC determination.

### Metabonomics analysis

#### Sample analysis

In this experiment, the ESI-MS method combining positive and negative ion detection modes will be used to treat urine and feces accordingly using the above pre-treatment methods, and the liquid chromatography conditions and mass spectrometry (MS) detection parameters will be optimized with a scanning range of m/z50-1000 to maximize the coverage of the metabolite groups. The goal is to establish an RP/HILIC-UPLC-TOFMS technique for analyzing various metabolites in urine and feces samples. 20 µL and 1 g of all test samples will be absorbed and thoroughly mixed in the entire analysis batch to be quality control (QC) samples.

#### Data preprocessing

The original atlas data obtained from the analysis system underwent preprocessing, followed by multivariate statistical analysis using MarkerView™ software. The primary processing steps involved setting appropriate parameters for retention time, noise threshold, minimum chromatographic peak width, and allowable error of molecular weight to perform background subtraction, chromatographic peak search, and calibration on the raw chromatographic data. The “80% rule” is applied to eliminate missing values. Additionally, if necessary, the analysis data could be normalized using the “always available mass spectrometry signal method” and “osmotic pressure normalization method”.

#### Multivariate Statistical Analysis

Preprocessed data will be subjected to unsupervised data analysis using Principal Component Analysis (PCA). A score plot will be used to observe clustering of data from different groups and to remove outlier samples. A loading plot will be utilized to identify components that contribute to inter-group variability, with special attention given to excluding prototype compounds and their metabolites. Additionally, univariate analysis such as fold change (FC) and t-tests will be performed to identify components that exhibit significant statistical differences. Pattern recognition methods, such as Partial Least Squares Discriminant Analysis (PLS-DA) and Orthogonal Partial Least Squares Discriminant Analysis (OPLS-DA), will be employed, and targeted programming and execution using languages such as R or C will be based on the characteristics of the analysis data. Integration of HILIC-TOFMS and RPLC-TOFMS data will be conducted to identify differential components. Multiple methods, including t-tests (*p* < 0.05), FC analysis (FC > 2), and PLS-DA (VIP > 1.5), will be used to screen and confirm potential biomarkers that contribute to inter-group differences, thus identifying potential biomarkers associated with acupuncture treatment in children with TD.

#### Identification of potential biomarkers

Confirmation of potential biomarkers involves determining their retention time in the original chromatogram, as well as utilizing accurate molecular weight, isotope distribution, and ion information (parent ions, fragment ions, adduct ions, and multiply charged ions) provided by TOFMS. This information, along with assistance from the PeakView™ software, aids in calculating the possible molecular formula. Preliminary identification of potential biomarkers is conducted by searching databases such as ChemSpide, MassBank, MetFrag, and Metlin. Finally, the identification is confirmed by using corresponding reference standards.

#### Metabolic pathway analysis

Through the analysis of the correlation between the levels of potential biomarkers and the scores of related scales, we identified relevant biomarkers in acupuncture treatment for children with TD. Additionally, we examined the KEGG database to identify metabolic pathways associated with these biomarkers in acupuncture treatment for TD children. Drawing upon this information, we constructed physiological and biochemical metabolic pathways in vivo related to the biomarkers, thus providing insights into the mechanism of acupuncture treatment for TD children.

## Observation time point

### Main symptoms and signs

Observations and recordings will be made during the initial visit on the first day and during the follow-up visit at the 12th week.

### Other indicators

Observations and recordings will be made during the initial visit on the first day and during the follow-up visit at the 12th week.

## Adverse events

Various adverse reactions that may occur during acupuncture treatment include dizziness, bleeding, infection, low fever, and organ damage. Infrared thermal imaging is a harmless and non-invasive method that poses no radiation risk to children. However, it requires exposing the back area during imaging, which may increase the risk of children catching a cold. The collection of feces may frighten children, leading to crying. Allergic reactions may occur in the control group when the acupuncture needle comes into contact with the skin during sham acupuncture. Additionally, sham acupuncture may cause pain, causing children to cry out of fear and discomfort. All adverse reactions observed during the study will be truthfully recorded.

## Quality control

The YGTSS scale will be assessed by a qualified physician. Standard operating procedures and quality control procedures will be established for observing indicators using infrared thermal imaging, with computer-generated results and traceable data and images. We will analyze and process the data through professional instruments to ensure the accuracy of the data. Prior to the project commencement, researchers received training on the experimental protocol, and the consistency of quantitative symptom and sign assessment standards was tested. Researchers signed a statement of commitment. Researchers should diligently obtain informed consent to ensure that participants fully understand the experimental requirements and are willing to cooperate. Treatment compliance of patients in the experimental group is monitored using the treatment count method. Compliance is calculated as the actual number of treatments received divided by the initially scheduled number of treatments, multiplied by 100%. Compliance rates below 80% or above 120% are considered major violations of the experimental protocol. The results before and after treatment should be communicated to the families of the patients.

## Data management and monitoring

The researchers will use research medical records to collect data and establish a database. The recorded data in the research medical records will be summarized and analyzed to generate the final conclusions and research report. Upon completion of the study, the database will be locked for security purposes. The personal information of the participants will be treated as confidential. Documents related to quality control, such as original records of data consistency check, numerical range and logic check, and blind audit, will be preserved. Meanwhile, we have trained researchers to establish a good communication relationship with patients. For patients who discontinue or deviate from intervention protocols, we will try to get as much trial data as possible by telephone, if the patient is willing to provide it. In addition, a safety monitoring committee will be established to review and interpret the data of trial. They will review the progress of the trial, independently of the investigators, and decide whether the trial needs to be terminated early solely on the basis of adverse events.

## Statistical analysis

Descriptive statistical analysis, qualitative indicators are described by frequency table, percentage or composition ratio; Quantitative indicators are described by mean, standard deviation, or median, lower quartile (Q1), upper quartile (Q3), minimum value and maximum value.

The qualitative data are analyzed using the chi-square test, Fisher’s exact probability method, Wilcoxon rank sum test, CMH χ2 test, and WLS covariance. For the quantitative data, the normal distribution is verified using the t test (homogeneity test of variance is conducted between groups, with a test level of 0.05, and the Satterthwaite method is used for correction when the variance is uneven). For non-normal distribution, the Wilcoxon rank sum test and Wilcoxon signed rank sum test are employed, along with GLM covariance. A two-sided test is uniformly used for the hypothesis testing, and test statistics along with their corresponding P-values are provided, with *P* ≤ 0.05 indicating statistical significance and *P* ≤ 0.01 indicating high statistical significance.

## Processing of infrared thermographic images

The temperatures of specific acupoints (Dazhui, Zhiyang, Mingmen, Yaoyangguan, and tendon contraction) in the area of the Du meridian will be manually traced. Use SPSS 20.0 statistical software for analysis. Quantitative data is expressed as “mean ± standard deviation” (x ± s). If the data follows a normal distribution, paired sample t-test is used; if the data does not follow a normal distribution, non-parametric tests (Wilcoxon signed-rank test) are used. Skin temperature comparison before and after intervention at different acupoints in different groups is analyzed using one-way analysis of variance (ANOVA) if it follows a normal distribution and has equal variances. If the data follows a normal distribution but has unequal variances, Welch’s test is used. If the data does not follow a normal distribution, Kruskal-Wallis H test is used. *P* < 0.05 indicates statistical significance of the differences.

## Discussion

Currently, the clinical diagnosis of TIC disorder (TD) is primarily based on scale evaluation, which lacks of objective evaluation indicators and detection methods. However, there are metabolic differences in urine and feces between children with TD and healthy children. In order to further investigate the pathogenesis of TD, it is crucial to explore the urine and feces metabonomic analysis. In our preliminary study, our research discovered a distinct difference in the body temperature along the governor vessel between children with TD and healthy children: the heat distribution along the governor vessel of children with TD is in uneven state when compared to that of healthy children. Importantly, this uneven heat distribution is significantly improved after acupuncture treatment. Therefore, we hypothesize that this metabolic abnormality may be related to the changes of structure of infrared thermography of specific areas along the governor vessel and acupoints around it. Since currently, there is no effective diagnostic equipment for TD, so by fully utilizing the infrared thermal imaging technology and metabonomic analysis to further explore the underlying molecular mechanism by which changes of body temperature occur holds potential in identifying targets for future research purpose on the mechanism of acupuncture treatment aimed at regulating energy metabolism in children with TD.

This approach may also aid in the identification of micro-biomarkers associated with treatment outcomes and contribute to the development of a theoretical and experimental basis for diagnosing TD using infrared thermal imaging technology. However, this experiment has several limitations, including a small sample size, a long treatment cycle, low cooperation of children, and a potentially high dropout rate. Consequently, further studies with larger sample sizes, longer-term interventions, and comprehensive follow-up evaluations are needed.

### Trial status

Protocol: version 3.0, March 16th, 2023. The participants are currently being recruited for the present study.

### Electronic supplementary material

Below is the link to the electronic supplementary material.

Supplementary Material 1

Supplementary Material 2

## Data Availability

The datasets generated and analysed during the current study are not publicly available due to the protection of subject privacy, but are available from the corresponding author on reasonable request.

## Abbreviations

TD: Tic disorder
TTM: Thermal Texture Maps
CSF: cerebrospinal fluid
cAMP: cyclic adenosine monophosphate
CRH: corticotropin-releasing hormone
YGTSS: Yale Comprehensive Tic Severity Scale
