# Supplementary material for: Effect of acupuncture on tic disorder: a randomized controlled clinical trial based on energy metabolomics and infrared thermography
Source: BMC Complement Med Ther. 2024 Jun 20;24:240. doi: 10.1186/s12906-024-04534-x (PMC11191346; doi:10.1186/s12906-024-04534-x)
Supplement: Supplementary file 1 — Supplementary Material 1 [file 12906_2024_4534_MOESM1_ESM.docx]

All acupuncture treatments conform to the Report Standard of Intervention Measures in Acupuncture Clinical Trials.

| **Item** | **Detail** | **Intervention** |
| --- | --- | --- |
| Acupuncture rationale | 1a Style of acupuncture | Traditional needle acupuncture |
|  | 1bReasoning for treatment provided, based on historical context, literature sources, and/or consensus methods, with references where appropriate | Based on traditional Chinese medicine theory and clinical experience, tic disorder is believed to be associated with an imbalance of Yang energy due to hyperactivity of Liver Yang. Therefore, the following acupoints will be selected，such as DU20、DU14、EX-HN3 and bilateral BL18、LI4 、ST36、SP6、LR3。 |
|  | 1cExtent to which treatment was varied | No variation |
| Details of needling | 2a Number of needle insertions per subject per session (mean and range where relevant) | thirteen needles insertion per subject per session |
|  | 2b Names (or location if no standard name) of points used (uni/bilateral) | DU20、DU14、EX-HN3 and bilateral BL18、LI4 、ST36、SP6、LR3 |
|  | 2c Depth of insertion, based on a specified unit of measurement, or on a particular tissue level | From 12.5 to 25 mm |
|  | 2d Response sought (e.g.de qi or muscle twitch response) | Deqi (a composite of sensations including soreness, numbness, distention, heaviness, and other sensations) |
|  | 2e Needle stimulation (e.g. manual, electrical) | electrical stimulation（continuous wave） |
|  | 2f Needle retention time | 30 min |
|  | 2gNeedle type (diameter, length, and manufacturer or material) | 0.30 mm × 25 mm disposable sterile needles（Huatuo brand, Suzhou, China） |
| Treatment regimen | 3a Number of treatment sessions | 36 sessions in total |
|  | 3b Frequency and duration of treatment sessions | three sessions per week for 12 consecutive weeks |
| Other components of treatment | 4a Details of other interventions administered to the acupuncture group (e.g. moxibustion, cupping, herbs, exercises, lifestyle advice) | Two groups will receive behavioral therapy. |
|  | 4b Setting and context of treatment, including instructions to practitioners, and information and explanations to patients | All practitioners received a 3-day training session before study initiation. |
| Practitioner background | 5 Description of participating acupuncturists (qualification or professional affiliation, years in acupuncture practice, other relevant experience) | All acupuncturists who have a license and at least 3 years of acupuncture practice |
| Control or comparator interventions | 6a Rationale for the control or comparator in the context of the research question, with sources that justify this choice | Sham acupuncture |
|  | 6b Precise description of the control or comparator. If sham acupuncture or any other type of acupuncture-like control is used, provide details as for Items l to 3 above | Same as intervention group except 2c) ,2d) and2g)  2c) The needle body and the needle handle are not fixed and can slide relatively freely, and the needle tip is round and blunt to simulate the acupuncture sensation of the real acupuncture needle inserted into the skin, and the needle tip does not penetrate the skin.  2d) Without Deqi sensation  2g) Φ0.30×25mmDisposable sterile blunt needle(Acu Prime brand, Dong Bang Acupuncture company, Britain) |
